# The Zintl–Klemm Concept in the Amorphous State: A Case Study of Na–P Battery Anodes

**DOI:** 10.1002/anie.202508305

**Published:** 2025-12-18

**Authors:** Litong Wu, Volker L. Deringer

**Affiliations:** ^1^ Inorganic Chemistry Laboratory Department of Chemistry University of Oxford Oxford OX1 3QR United Kingdom

**Keywords:** Battery materials, Computational chemistry, Interatomic potentials, Machine learning, Phosphorus

## Abstract

The Zintl–Klemm concept has long been used to explain and predict the bonding, and thereby the structures, of crystalline solid‐state materials. We apply this concept to the amorphous state, examining as an example the diverse disordered Na–P phases that can form in sodium‐ion battery anodes. Using first‐principles computations combined with state‐of‐the‐art machine‐learning methods, we provide atomic‐scale insights into the structures and energetics of amorphous Na–P phases. We showcase the applicability of the Zintl–Klemm rules in the amorphous state and discuss implications for future work.

## Graphical abstract

The long‐established Zintl–Klemm concept can be applied to amorphous materials just as well — as is shown here for disordered Na–P phases that are relevant for sodium‐ion battery anodes. Machine‐learning‐driven, quantum‐mechanically accurate simulations yiel insights into structure and bonding, and unlock the atomistic modeling of structural changes during battery operation.

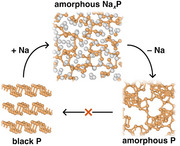

Since its early foundation in the 1930s, the Zintl–Klemm concept has remained a powerful framework for describing the bonding and structure of compounds formed between electropositive alkali and alkaline‐earth metals, and electronegative elements from groups 13 to 16 of the periodic table.^[^ ^1^, ^2^ ^]^ In this concept, the alkali or alkaline‐earth metals formally donate their valence electrons to the more electronegative p‐block elements, which in turn achieve closed‐shell electronic configurations by accepting the transferred electrons and forming covalent bonds among themselves.^[^ ^3^ ^]^ As a result, Zintl phases exhibit salt‐like characteristics due to the ionic interactions between cations and polyanionic units.^[^ ^4^ ^]^ In the 1950s, Klemm further built on Zintl's idea by introducing *the pseudoatom model*, in which the anionic units are treated as elements with the same number of valence electrons.^[^ ^5^, ^6^ ^]^ Klemm's work set the stage for iso‐ and aliovalent modifications in materials design, enabling the guided synthesis of pseudoatom‐inspired compounds.^[^ ^1^, ^3^ ^]^

Zintl phases span the spectrum between classical salts and intermetallic compounds, encompassing materials with a diverse range of structural and electronic properties.^[^ ^2^, ^7^ ^]^ Various subclasses, including both ordered crystalline phases and those exhibiting correlated disorder, have been extensively studied for different applications. For instance, structurally disordered Zintl phases have been widely investigated in the context of thermoelectric materials, where the presence of defects and disordered substructures can be crucial for tuning lattice thermal conductivity and electronic transport.^[^ ^8^, ^9^ ^]^ Despite their lack of long‐range order, these systems typically yield diffraction patterns with well‐defined Bragg peaks.^[^ ^10^, ^11^ ^]^ Fully amorphous Zintl phases remain largely unexplored, with only occasional reports and few systematic studies to date.^[^ ^1^ ^]^

An area in which Zintl‐phase amorphization is frequently observed is alloy‐based battery anodes, typically group‐14 (Si,^[^ ^12^ ^]^ Ge,^[^ ^12^ ^]^ Sn^[^ ^13^, ^14^ ^]^) or group‐15 (P, Sb^[^ ^15^, ^16^ ^]^) elements, which store Li, Na, or K ions through binary alloying or conversion reactions. Unlike durable intercalation‐based electrode systems, these materials undergo repeated structural destruction and reconstruction during metal insertion and extraction.^[^ ^17^ ^]^ While offering high theoretical capacities and low discharge voltages, their practical application is often limited by substantial volume expansion and unstable interfaces.^[^ ^18^ ^]^ Understanding the mechanisms during alloy‐based anode operation is therefore crucial, especially for Na‐ion batteries (SIBs) which are considered a more cost‐effective and sustainable alternative to Li‐ion batteries, but still face challenges in anode design. In particular, the larger ionic radius of Na prevents efficient intercalation into commercial graphite anodes, necessitating the search for alternative high‐capacity anode materials.^[^ ^17^ ^]^

Here, we focus on phosphorus, a promising SIB anode material with a theoretical capacity of 2 596 mA h $g^{-1}$ for its fully sodiated phase, ${\mathrm{Na}}_{3}P$.^[^ ^19^ ^]^ Both crystalline black and amorphous red P have been extensively investigated experimentally as SIB anode materials. Black P is a semiconducting “van der Waals” material with puckered six‐membered rings within its layers.^[^ ^20^, ^21^ ^]^ The commercially available red P, in contrast, comprises diverse cluster fragments primarily formed from interconnected five‐ and six‐membered rings.^[^ ^22^ ^]^ These local structural motifs closely resemble those found in violet and fibrous P^[^ ^23^, ^24^ ^]^ as well as P nanorods.^[^ ^25^, ^26^ ^]^ Bulk and monolayer black P^[^ ^27^, ^28^, ^29^ ^]^ as well as bulk red P^[^ ^19^, ^30^, ^31^, ^32^ ^]^ have been integrated with conductive carbon materials, forming nanostructured composites that showed excellent electrochemical performance in SIBs. While carbon materials contribute minimally to the capacity, they serve as a mechanical backbone and an electron‐conducting matrix, enhancing cycling stability. Notably, most studies have identified amorphous Na–P (a‐${\mathrm{Na}}_{x}P$) phases as key intermediates during battery cycling.^[^ ^19^, ^27^, ^28^, ^29^ ^]^

To address the challenge of understanding these amorphous phases at the atomic scale, machine‐learning methods provide a powerful tool by enabling efficient first‐principles atomistic simulations.^[^ ^33^, ^34^, ^35^ ^]^ Machine‐learning‐based interatomic potential (MLIP) models have been applied to elemental P to describe the phase transition between molecular and network liquid forms,^[^ ^36^ ^]^ explore hypothetical hierarchically‐structured allotropes,^[^ ^37^ ^]^ and study the structural and bonding characteristics of amorphous red P.^[^ ^22^, ^38^ ^]^ Building on these advances, our present work moves beyond the elemental system and explores the structural and energetic landscape of a‐${\mathrm{Na}}_{x}P$ compounds. Our simulations are based on a custom‐fitted MLIP model developed using the MACE architecture,^[^ ^39^ ^]^ which achieves root‐mean‐square errors of 7.7 meV at.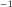 for energy and 0.13 eV Å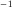 for force predictions (Figure S2). Previous work suggests that short‐range MLIPs could accurately describe isotropic bulk systems without explicit long‐range terms;^[^ ^40^, ^41^ ^]^ therefore, the message‐passing design of MACE, with its additional advantage in capturing longer‐range interactions, seems well‐suited for modeling the Na–P system. Full details of the computational approach, including dataset construction and training protocols, are provided in the Supporting Information.^[^ ^42^ ^]^

To systematically explore the compositional range from elemental P to ${\mathrm{Na}}_{3}P$, we performed ML‐driven melt–quench molecular‐dynamics (MD) simulations to generate a series of a‐${\mathrm{Na}}_{x}P$ structures — each containing 248 P atoms with varying Na content, up to a maximum of 744 Na atoms (x = 3). Initially, pristine a‐P structures were generated following established protocols.^[^ ^22^, ^38^ ^]^ To accommodate Na atoms within the covalent P frameworks, it was necessary to expand the a‐P cells: target volumes per P atom for different Na content were estimated via linear least‐squares regression of crystalline‐phase volumes (Supporting Information). After isotropically expanding the cells to the calculated volumes, Na atoms were inserted at random positions with a hard‐sphere cutoff. The resulting structures were melted at 1 200 K and quenched at a rate of 10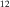 K $s^{-1}$ to obtain amorphous structure models. Selected a‐P and a‐${\mathrm{Na}}_{x}P$ structures are shown in Figure 1a, using smaller 108‐P systems for visual clarity. Na atoms are shown as grey spheres, while P atoms are color‐coded according to their homonuclear coordination number, N. From left to right, as the Na content increases, the P frameworks obtained in the simulations range from extended networks to branched chains, chain fragments, and eventually isolated ions.

**Figure 1:**
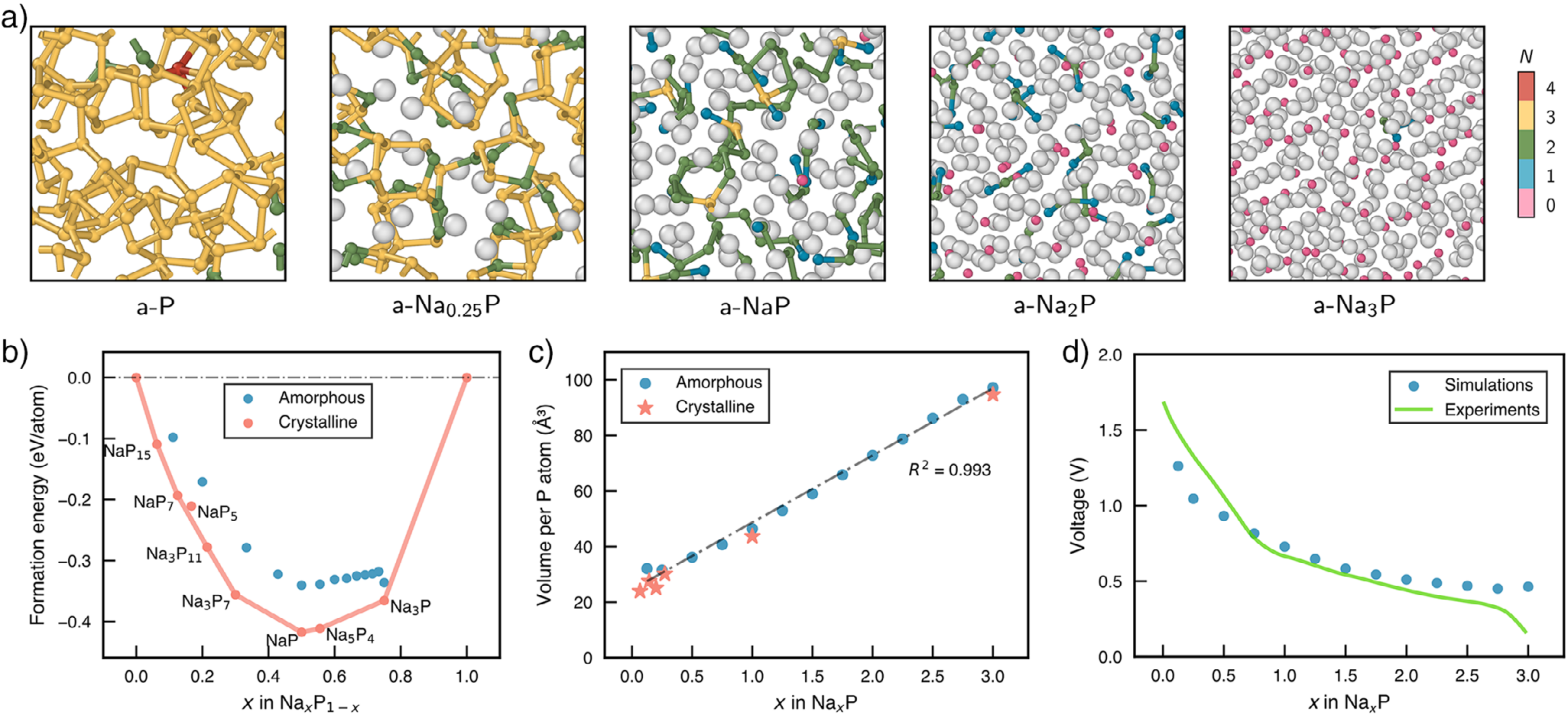
Amorphous Na–P phases from ML‐driven simulations. a) Representative structures of a‐P and a‐${\mathrm{Na}}_{x}P$ obtained from melt–quench simulations, shown in order of increasing Na content (grey spheres). P atoms are color‐coded by the number of other P atoms to which they are bonded: N=4 (red), N=3 (yellow), N=2 (green), N=1 (blue), and N=0 (pink), determined using a 2.4 Å cutoff. The cell images have been cropped for better visualization, and hence the borders do not correspond to the actual simulation cell edges. b) Normalized formation energies as a function of fractional Na content in Na–P compounds, referenced to black P and body‐centered cubic Na. The convex hull is constructed by joining stable crystalline Na–P phases (red); amorphous phases are shown in blue. DFT energies are used for crystalline structures; MACE energies for amorphous structures. c) Simulation cell volumes normalized by the number of P atoms, plotted against Na content. A best‐fit line for the amorphous‐phase volumes is shown. d) Voltage profile for the sodiation of amorphous P. Experimental data from Capone et al. (Ref. [30]) are shown by a green line.

To assess the thermodynamic stability of the amorphous phases, we constructed the convex hull using the formation energies of stable crystalline Na–P phases (Figure 1b), calculated from DFT energies akin to Ref. [43]. All on‐hull phases have been experimentally observed, with the exception of ${\mathrm{Na}}_{5}P$ 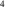, which was theoretically predicted to be stable.^[^ ^43^ ^]^ The known ${\mathrm{NaP}}_{5}$ phase,^[^ ^44^ ^]^ although located above the computed convex hull, is included for comparison. Formation energies of the amorphous samples, calculated using our MACE model, are plotted relative to the convex hull. As expected, the a‐${\mathrm{Na}}_{x}P$ phases lie above the hull, reflecting their lower thermodynamic stability compared to crystalline structures. Notably, our a‐${\mathrm{Na}}_{3}P$ sample exhibits an anomalous stabilization, likely suggesting a tendency to crystallize.

Since structural expansion is a key challenge that limits the longevity of P anodes, simulation cell volumes normalized by the number of P atoms are plotted in Figure 1c. The data reveal an almost linear increase in normalized volume as the number of Na atoms increases, with a strong correlation ($R^{2}=0.993$). The volumes of the amorphous phases are comparable to those of their crystalline counterparts, and including both crystalline and amorphous structures in a linear regression reduces the $R^{2}$ value by less than 0.001. In particular, the a‐${\mathrm{Na}}_{3}P$ structure expands by 329% relative to the initial a‐P structure, closely matching the theoretical prediction of 331%.^[^ ^30^ ^]^

Another experimentally relevant quantity, the voltage at different stages of sodiation, was estimated computationally similar to previous work^[^ ^45^ ^]^ (Figure 1d). An experimental charging profile of Na with ball‐milled red‐P–graphite electrodes, from Ref. [30], is plotted in green for comparison. A reasonably good agreement is seen in the intermediate composition range, supporting the validity of the MLIP model. However, it is important to note that experimentally measured voltages can be affected by side reactions and kinetic limitations, not captured on the time scale of typical MD simulations. The selected experimental data, based on steady‐state potentials from galvanostatic intermittent titration technique measurements,^[^ ^46^ ^]^ offer a more reliable estimate of the true equilibrium potential across different states of charge.

We next examine the applicability of the Zintl–Klemm concept to a‐${\mathrm{Na}}_{x}P$ structures in Figure 2. In this analysis, P atoms in all a‐${\mathrm{Na}}_{x}P$ structures are classified and color‐coded according to their respective homonuclear connectivity; the labels (0b), (1b), (2b), and (3b) denote P atoms with zero, one, two, or three P–P bonds. Formal charges are then assigned as superscripts based on the valence‐electron requirements. According to the Zintl–Klemm concept, two key implications arise: first, the total formal oxidation state of the P framework should balance out that of the Na atoms in each a‐${\mathrm{Na}}_{x}P$ structure; second, the resulting ‘pseudoelements’ formed by P after the electron transfer may adopt structural motifs analogous to those of their isoelectronic counterparts.

**Figure 2:**
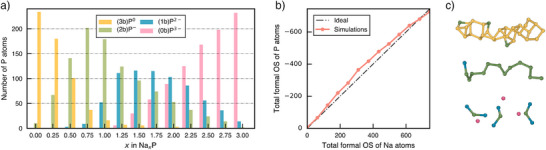
The Zintl–Klemm concept applied to amorphous Na–P phases. a) Distribution of P atoms with different homoatomic coordination numbers in each a‐${\mathrm{Na}}_{x}P$ configuration across the compositional range. P atoms are classified by a connectivity symbol in parentheses and the formal charge as superscript. b) Total formal oxidation state (OS) of P atoms compared to Na atoms in each a‐${\mathrm{Na}}_{x}P$ structure. The ideal values are shown by a dash‐dotted line. c) Selected local structural fragments from a‐${\mathrm{Na}}_{\text{0.125}}P$, a‐NaP, and a‐${\mathrm{Na}}_{2}P$ (top to bottom).

Figure 2a shows the distribution of P atoms in each connectivity category across the compositional range in the amorphous structures. As expected, with increasing Na content, greater proportions of P atoms exhibit lower homonuclear connectivity, indicating P–P covalent bond breaking induced by Na–P interactions. This trend aligns with the chemical expectation that electron donation from Na atoms reduces the need for P–P bonding to satisfy valence requirements. Interestingly, the proportion of (1b)$P^{2-}$ species remains comparatively low across all compositions, even in regimes where it would be expected to dominate. In addition, the corresponding total formal oxidation state for each configuration, shown in Figure 2b, conforms well to the ideal Zintl–Klemm formulation indicated by a dash‐dotted line. This implies that simple local bonding rules, when coupled with the high configurational flexibility of P atoms, can give rise to complex amorphous networks.

Representative local structural fragments from our simulations are shown in Figure 2c. The top fragment, taken from an a‐${\mathrm{Na}}_{0.125}P$ cell, exhibits structural features reminiscent of a‐P, with clusters composed of interconnected five‐ and six‐membered rings.^[^ ^38^, ^47^, ^48^ ^]^ Within these clusters, (2b)$P^{-}$ ions are often found near Na atoms — an arrangement also observed in the mixed‐valence polymeric P framework of crystalline ${\mathrm{NaP}}_{15}$ ^[^ ^49^ ^]^ and ${\mathrm{NaP}}_{7}$ ^[^ ^50^ ^]^ (Figure S12). As sodiation proceeds, these clusters gradually break apart, giving rise to chain‐like motifs, that dominate near the NaP stoichiometry. Compared to the helical chain found in crystalline NaP,^[^ ^51^ ^]^ chains in the amorphous structures do not exhibit local symmetry, with occasional branching and termination. This transition in the dominant structural motifs highlights the second key implication of the Zintl–Klemm concept: in a‐NaP, the formally (2b)$P^{-}$ ions are isoelectronic to S atoms. Indeed, chain‐like motifs are common in the allotropes of S,^[^ ^52^ ^]^ Se,^[^ ^53^ ^]^ and Te.^[^ ^54^, ^55^ ^]^

Upon further sodiation, these chains progressively fragment. In a‐${\mathrm{Na}}_{2}P$, the (1b)$P^{2-}$ ions, isoelectronic to Cl, might be expected to form dumbbells with isolobal analogy to ${\mathrm{Cl}}_{2}$ molecules (cf. Ref. [56]). However, such dumbbell‐like motifs are not prevalent in the a‐${\mathrm{Na}}_{2}P$ samples (Figure 1a). Instead, the dominant structural motifs at this composition are bent triatomic molecules, accompanied by isolated (0b)$P^{3-}$ ions, as shown at the bottom of Figure 2c. This trimer species, (1b)$P^{2-}$–(2b)$P^{-}$–(1b)$P^{2-}$, is both isoelectronic and isostructural to the well‐known ${\mathrm{SCl}}_{2}$ molecule. Principally, two dumbbell units are electronically equivalent to one bent trimer and one isolated phosphide anion, and the interconversion between these species can theoretically be achieved via a (formal) disproportionation reaction:

**Table:** 

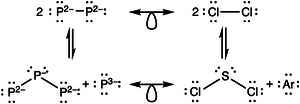

To understand the underrepresentation of (1b)$P^{2-}$ and the preference for ${\mathrm{SCl}}_{2}$‐like trimers over ${\mathrm{Cl}}_{2}$‐like dumbbells, we analyzed the MACE‐predicted local *per‐atom* energy distributions for P atoms with different local connectivity in all a‐${\mathrm{Na}}_{x}P$ structures, as shown in Figure 3. Although the interpretability of MLIP atomic energies remains a topic of debate, growing evidence suggests that these local energy values can provide meaningful insights.^[^ ^57^, ^58^, ^59^, ^60^ ^]^ Here, we observe that the average atomic energy of (1b)$P^{2-}$ exceeds that of (0b)$P^{3-}$, and is in fact the highest among all four P environments in a‐${\mathrm{Na}}_{x}P$. This observation may help explain the preferential formation of bent trimers with isolated phosphide anions over dumbbells: despite having the same overall formal oxidation state, the former incurs a lower energetic cost by having two (1b)$P^{2-}$ ions instead of four.

**Figure 3:**
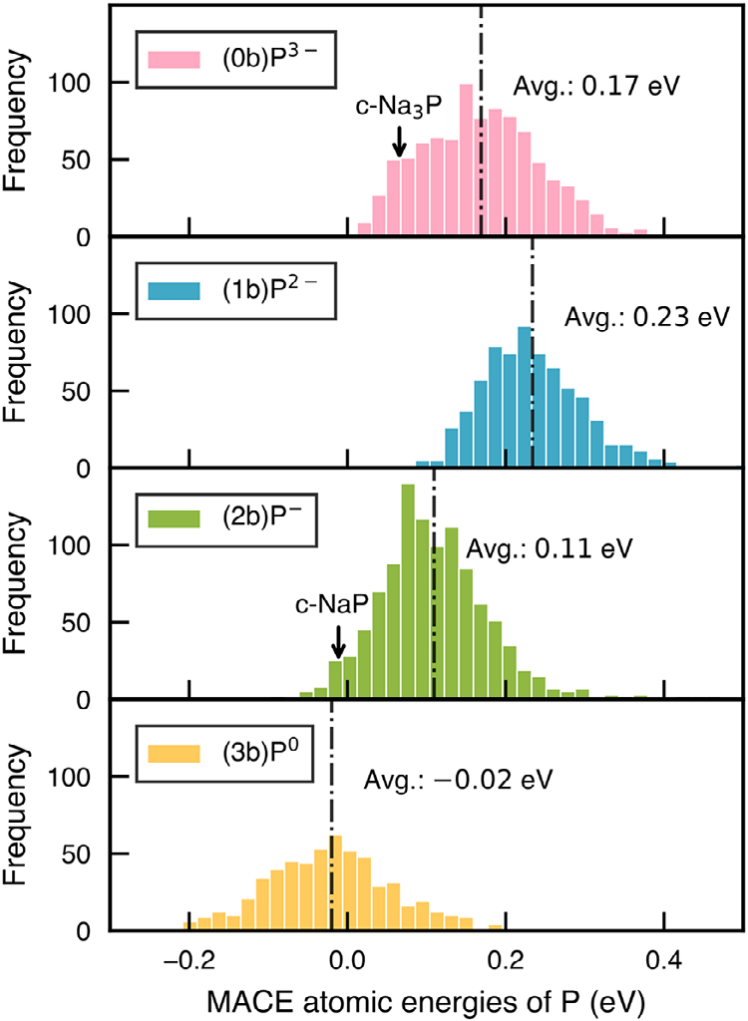
Distributions of the MACE atomic energies of P atoms in a‐${\mathrm{Na}}_{x}P$ (0.125≤x≤3) structures, shown separately for P atoms with different local connectivity. All values are referenced to the atomic energy of crystalline black P. The atomic energies of P atoms in crystalline ${\mathrm{Na}}_{3}P$ and NaP are marked with arrows. The average atomic energy for each category is indicated by vertical dash‐dotted lines. A similar analysis for Na atoms is discussed in the Supporting Information.

The instability of dumbbell‐like motifs is also consistent with previous experimental and computational findings. 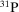 NMR study by Marbella et al. suggested that dumbbell‐containing structures were not present in significant quantities in SIBs during cycling.^[^ ^29^ ^]^ Similarly, ab initio studies of Li‐ and Na‐ion anode materials by Mayo et al. revealed that while P dumbbells are common in ${\mathrm{Li}}_{x}P$ when 1.33<x≤2, they are absent in the Na–P system.^[^ ^43^ ^]^ The same study identified ${\mathrm{Na}}_{4}P$ 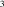, a locally stable structure only 2 meV per formula unit above the convex hull, as comprising bent P trimers similar to those observed in our amorphous structures. Both aforementioned studies employed random structure searching, in which many small Na–P unit cells were generated under physical constraints and relaxed with DFT to identify low‐energy configurations near the convex hull. In contrast, in this work, amorphous structures were generated using a melt–quench protocol combined with an MLIP, which captures realistic temperature‐ and pressure‐dependent atomic responses, and enables simulations at length scales that better represent disordered phases. Despite these methodological differences, similar atomic behaviors were observed.

To further understand the local behavior of P atoms, atomic charges were computed using the Löwdin scheme^[^ ^61^ ^]^ as implemented in the LOBSTER software.^[^ ^62^, ^63^, ^64^ ^]^ An independent set of smaller a‐${\mathrm{Na}}_{x}P$ structures (each containing 108 P atoms) was generated following the same melt–quench protocol to perform the charge calculations. Table 1 summarizes the Löwdin charges of P atoms with different local coordination: as expected, P species with more negative formal oxidation states based on the coordination analysis also exhibit more negative average Löwdin charges. The relatively small standard deviations compared to the differences in mean values suggest that the charge distributions within each coordination class are fairly narrow, indicating a clear differentiation across bonding environments.

**Table 1:** Average Löwdin charge and corresponding standard deviation for P atoms with different local connectivity in 108‐P a‐${\mathrm{Na}}_{x}P$ (0.25≤x≤3) structures. A similar analysis for Na is discussed in the Supporting Information.

Coordination	Charge (e)
(0b)$P^{3-}$	−1.447 ± 0.031
(1b)$P^{2-}$	−0.984 ± 0.060
(2b)$P^{-}$	−0.470 ± 0.100
(3b)$P^{0}$	−0.066 ± 0.040

We note that the magnitude of Löwdin charges is consistently smaller than that of the formal charges. This difference is to be expected: the Zintl model is a valence‐based heuristic assuming full electron transfer, which can lead to overestimated charges; Löwdin charges are derived from quantum‐mechanical orbital projections, which are reported to systematically underestimate ionic character in strongly ionic systems.^[^ ^65^ ^]^ And yet, the charge analysis provides a quantitative complement to the Zintl–Klemm concept — it confirms the electronic distinction between different P coordination classes, and highlights the increasingly ionic character of P species in a‐${\mathrm{Na}}_{x}P$ structures with increasing Na content.

Finally, a series of small‐scale, proof‐of‐concept MACE‐driven MD simulations were carried out to investigate the structural evolution during sodiation and de‐sodiation, inspired by earlier ab initio MD studies.^[^ ^66^ ^]^ The initial configuration of the simulation (Figure 4a) was based on a black P supercell containing four layers (256 P atoms), positioned along one side of an elongated simulation cell. An equal number of Na atoms were randomly distributed in the adjacent space along the x‐direction. This orientation was chosen because only channels along the x‐axis provide sufficient width (3.08 Å) to accommodate the diffusion of Na ions (diameter =2.04 Å^[^ ^67^ ^]^), whereas channels along the y‐axis are significantly narrower (1.16 Å) and therefore inaccessible.^[^ ^27^ ^]^

**Figure 4:**
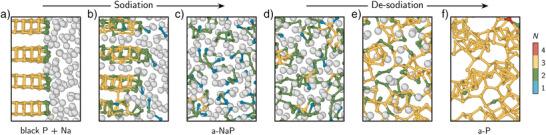
Structural change of black P during sodiation and de‐sodiation. a) Initial configuration of the simulation cell, with a 256‐atom black P supercell positioned on the left and Na atoms randomly placed in the adjacent space on the right. b) Intermediate configuration during sodiation, showing Na intercalation and partial P framework destruction. c) Final configuration of the 1:1 (Na:P) sodiation simulation. d)–f) Final configurations following the removal of 25%, 75%, and 100% of the Na atoms, respectively. The cell images have been cropped for visualization.

The sodiation simulation involved an NPT annealing at 600 K for 100 ps, followed by a quench. At elevated temperatures, molten Na atoms rapidly diffused into the van der Waals gaps between the layers, driven by the thermodynamics of Na–P interaction (Figure 4b). This Na infiltration initiated P–P bond rearrangements and breakage, eventually leading to the formation of a homogeneous amorphous Na–P phase (Figure 4c). To simulate the de‐sodiation process, Na atoms were systematically removed from the relaxed sodiated structure in four steps. Increments of 25% of the total Na content were extracted sequentially starting from atoms farthest from the originally P‐rich region along the x‐axis. After each extraction, the system was subjected to annealing at 600 K for 100 ps followed by quenching to allow for structural reorganization. This process was repeated four times until all Na atoms had been removed. The relaxed configurations after the removal of 25%, 75%, and 100% of Na atoms are displayed in Figure 4d–f.

During the simulation, progressive de‐sodiation led to a gradual reformation of P–P bonds. However, despite the reappearance of local bonding motifs, the original layered structure of black P was not recovered. Instead, the system evolved into an amorphous phase with a disordered network topology. Cluster analysis (Figure S10) reveals a substantial increase in the number of five‐membered rings and local cluster fragments, such as P3]P2[P3 and P2[P3]P2 Baudler units,^[^ ^47^, ^48^ ^]^ throughout the de‐sodiation process, while the number of six‐membered rings showed only a marginal increase. Since six‐membered rings are characteristic of crystalline black P, whereas five‐membered rings and local cluster fragments are commonly found in amorphous P,^[^ ^38^ ^]^ this observation suggests an irreversible amorphization of the P structure — in agreement with previous experimental reports of irreversible capacity loss and structural disorder in cycled P anodes.^[^ ^19^, ^27^, ^29^ ^]^

In conclusion, we have presented an ML‐driven approach for modeling and interpreting the complex chemical and structural features of the amorphous Na–P system. Coordination and charge analyses reveal that the local bonding environments in amorphous Na–P structures conform to the traditional Zintl–Klemm concept. Furthermore, our pilot simulations in Figure 4 captured diffusion‐driven structural evolution during (de‐) sodiation, providing insights into the irreversible amorphization of P anodes at the atomic scale, as well as a springboard for larger‐scale simulations. Extensions of this framework to related systems, such as Li–P or Na–Sb, appear conceptually promising and could be explored in future work. Future efforts could also focus on incorporating transport property characterization, with the long‐term goal of guiding the design and laboratory synthesis of improved anode materials.

## Conflict of Interests

The authors declare no conflict of interest.

## Supporting information

Supporting Information

## Data Availability

Data supporting this work are available at https://doi.org/10.5281/zenodo.15114751.
