# Supplementary material for: The Zintl–Klemm Concept in the Amorphous State: A Case Study of Na–P Battery Anodes
Source: Angew Chem Int Ed Engl. 2025 Dec 18;65(5):e08305. doi: 10.1002/anie.202508305 (PMC12851010; doi:10.1002/anie.202508305)
Supplement: Supplementary file 1 — Supporting Information [file ANIE-65-e08305-s001.pdf]

Supporting Information for

"The Zintl–Klemm Concept in the Amorphous State: A Case Study of Na–P Battery Anodes"

Litong Wu and Volker L. Deringer*

Inorganic Chemistry Laboratory, Department of Chemistry, University of Oxford, Oxford OX1 3QR, United Kingdom

Contents

S1 Computational methods **S2**

S1.1 Training dataset S2

S1.2 MACE training S7

S1.3 MD simulations S8

S1.4 DFT computations S10

S1.5 Charge and DOS computations S11

S1.6 Computational costs S11

S2 Supplementary results and discussions **S12**

S2.1 Effects of quench rates S12

S2.2 P–P bond lengths S13

S2.3 Atomic and environmental energies of Na atoms S14

S2.4 Atomic energies versus charges S15

S2.5 Electronic structure evolution with Na filling S15

S2.6 Ring and cluster fragment counts during de-sodiation S16

S2.7 Effects of many-body dispersion corrections S17

S2.8 Structural fragments in related crystalline phases S19

S3 Supplementary references **S20**

*volker.deringer@chem.ox.ac.uk

S1 Computational methods

S1.1 Training dataset

Our machine-learned interatomic potential (MLIP) model for Na–P phases was trained on a dataset developed over eight iterative training cycles. The present section outlines the creation and evolution of the training dataset.

S1.1.1 Iter-1.0

Table S1 presents the composition of the training dataset used for the first MACE model. Isolated Na and P atoms, as well as 64 dimers in vacuum were created in $20 \times 20 \times 20$ Å^3 simulation cells. The dimers were scaled to distances of 1.7 to 4.0 Å for Na–Na, 1.6 to 4.0 Å for Na–P, and 1.6 to 3.0 Å for P–P. The distorted crystalline P, liquid P, and GAP–RSS P structures were sourced and re-labelled from the P-GAP-20 dataset.[S1]

Table S1: Composition of the training dataset for Iter-1.0.

Configuration types	Number of structures	Number of atoms
Isolated atoms	2	2
Dimers	64	128
Amorphous P (melt–quench)	45	4,772
Amorphous Na_xP (geometry optimization)	405	56,848
Distorted crystalline Na	189	2,073
Distorted crystalline Na_xP	86	3,728
RSS Na	180	1,878
Distorted crystalline P	821	21,382
Liquid P	210	52,080
GAP–RSS P	302	5,286
**Total**	**2,304**	**148,177**

Amorphous P structures. Nine 108-atom amorphous P (a-P) structures were generated through melt–quench molecular dynamics (MD) simulations starting from an artificial cubic lattice containing 27 uniformly spaced tetrahedral P_4 molecules (P–P bond length = 2.2 Å), set to match the density of crystalline white P. This initial structure was melted into a molecular liquid via NPT simulations at 500 K and 1 bar for 20 ps. This was followed by compression from 0.3 GPa to 1.5 GPa at 2,000 K over 100 ps to induce network liquid formation, and quenching from 2,000 K to 0.1 K at a rate of 2×10^{13} K s^{-1} at 1 bar to form a-P structures. Low-density variants were created by isotropically expanding copies of each a-P structure to

densities of 2.0, 1.8, 1.6, and 1.4 g cm^{-3} respectively, melting in the NVT ensemble at 1,500 K for 50 ps, and quenching to 0.1 K at 10^{13} K s^{-1}. All MD simulations were performed using LAMMPS[S2] with a timestep of 1 fs. The resulting 45 a-P structures were included in Iter-1.0 training set and used for subsequent Na insertion.

Amorphous Na_xP structures. To create amorphous Na_xP (a-Na_xP) structures, Na atoms were randomly inserted into the previously made a-P frameworks while enforcing a hard-sphere cut-off distance of 2.2 Å for Na–P interactions and 2.4 Å for Na–Na interactions. Prior to Na insertion, any P_4 molecules that formed in the a-P cells during NVT melt–quench simulations of the expanded structures were removed. The number of Na atoms to insert varied based on the densities of the a-P structures, with 10 and 20 Na atoms added to a-P structures at 2.0 g cm^{-3}; 20, 30, and 40 at 1.8 g cm^{-3}; 40, 50, and 60 at 1.6 g cm^{-3}; and 60, 70, and 80 at 1.4 g cm^{-3}. In addition to the a-P structures, Na atoms were also inserted into a pristine 96-atom black P cell, generating 50 different structures with 5 to 50 Na atoms added each. All Na-inserted P structures were subsequently optimized using CASTEP,[S3] generating a total of 150 trajectories. Snapshots were extracted at 5%, 30%, and 100% of the optimization process from each trajectory, giving a total of 450 structures. Computational details of the geometry optimization can be found in Section S1.4.1.

Other structures. All elemental Na and binary Na–P structures reported in the Materials Project[S4] were included in the dataset. Angle- and volume-distorted variants of these structures were generated by applying a random distortion in the range of ±30% to all six lattice parameters, and 209 (Na) and 96 (Na–P) such structures were labelled, respectively. Additionally, 200 random Na structures were generated using the `buildcell` tool from the AIRSS package,[S5,S6] with target volumes ranging from 30 to 45 $Å^3$ per atom and a minimum atomic separation of 3.0 Å.

Structures in the dataset were labelled using density-functional theory (DFT) (Section S1.4.2), and were split such that 10% of each configuration type (excluding isolated atoms, dimers, and amorphous P structures) were reserved for testing, with the remainder used for training. Further details on MACE model training are provided in Section S1.2.

S1.1.2 Iter-1.1 to Iter-1.4

Using the Iter-1.0 MACE model, MD simulations were performed with the Atomic Simulation Environment (ASE) package[S7] to iteratively generate more a-Na_xP configurations. These iterative MD simulations aimed to expand both the compositional and configurational space by (i) increasing Na content up to a Na:P ratio of 3:1 and (ii) exploring the effects of elevated temperatures up to 1,500 K on a-Na_xP structures.

Amorphous Na_xP starting configurations were created using an expansion–insertion protocol similar to Section S1.1.1. Target volumes for a-P cell expansion were determined through trial and error guided by crystalline reference structures. The inserted structures were not further optimized but directly used in MACE MD simulations. In the initial simulations, a-Na_xP structures were heated from 300 K to 600 K over 10 ps and then equilibrated at 600 K for another 10 ps. From each MD trajectory, 20 snapshots were extracted at fixed intervals, labelled, and added into the training dataset for the next iteration of the MACE model. Once the simulations at lower temperatures stabilized, the updated MACE model was used for MD simulations at progressively higher temperatures (900 K, 1,200 K, and 1,500 K). These simulations were performed in the NVT ensemble using a Nosé–Hoover thermostat,[S8,S9] with a short 0.2 fs timestep to improve stability in MD for early potential versions.

S1.1.3 Iter-2.0

Several important changes were made in Iter-2.0:

Removal of SEDC. The Tkatchenko–Scheffler (TS) pairwise dispersion correction[S10] was initially employed for optimizing Na-inserted P structures and labelling datasets used in Iter-1 models (Section S1.4), given the known importance of dispersion interactions in accurately describing the elemental P system.[S1,S11,S12] However, as the Na content increased, it became apparent that the pairwise TS dispersion correction overestimated interactions between Na atoms, leading to unphysically short Na–Na distances and structural contraction in MD simulations. Similar issues have been reported when applying CASTEP's semiempirical dispersion correction (SEDC) to strongly ionic systems.[S13,S14] To address this issue, all configurations were re-labelled with SEDCs removed.

Removal of dimers. Dimers were removed from the training dataset, as a test indicated that their inclusion introduced notable numerical errors and led to unstable MD simulations.

Replacement of Na RSS structures. Na structures obtained from non-optimized random structure search (RSS) were replaced with GAP-driven RSS structures, following ideas and protocols described in Refs. S15 and S16, and created using the `autoplex` software.[S17] In this approach, 10,000 random structures, each containing 2 to 24 Na atoms, were initially generated with per-atom volumes of 30 to 45 Å^3 and a minimum interatomic separation of 3.0 Å. Each structure was labelled using a smooth overlap of atomic positions (SOAP) descriptor,[S18] and the CUR algorithm[S19] was applied to select the 100 most diverse structures. The selected structures were then subjected to single-point energy, force, and stress computations using VASP[S20] (Section S1.4.3) and split in a 9:1 ratio for training a Gaussian Approximation Potential (GAP).[S21] Iterative RSS proceeded such that this GAP potential was used to minimize the enthalpy of another set of 10,000 random structures. A combination of Boltzmann-biased flat histogram sampling and leverage-score CUR selection was then applied to preferentially select 100 lower-energy and structurally diverse configurations for the next iteration of GAP training. This process was repeated over ten iterations, and from the 1,000 structures generated, 250 were selected, labelled, and incorporated into the Iter-2.0 dataset.

Note that a few structures were lost during the re-labelling process due to incomplete CASTEP single-point computations.

S1.1.4 Iter-2.1 and Iter-2.2

Two additional rounds of iterative MD simulations were performed to generate more a-Na_xP structures. The composition of the training dataset used for the final production model, Iter-2.2, is shown in Table S2.

Table S2: Composition of the training dataset for the final production model (Iter-2.2).

Configuration types	Number of structures	Number of atoms
Isolated atoms	2	2
Amorphous P (melt–quench)	85	9,096
Amorphous Na_xP (geometry optimization)	390	54,290
Amorphous Na_xP (iterative MD)	575	124,522
Distorted crystalline Na	184	22,074
Distorted crystalline Na_xP	85	3,648
GAP–RSS Na	205	2,058
Distorted crystalline P	809	21,506
Liquid P	151	37,448
GAP–RSS P	297	5,508
**Total**	**2,783**	**260,152**

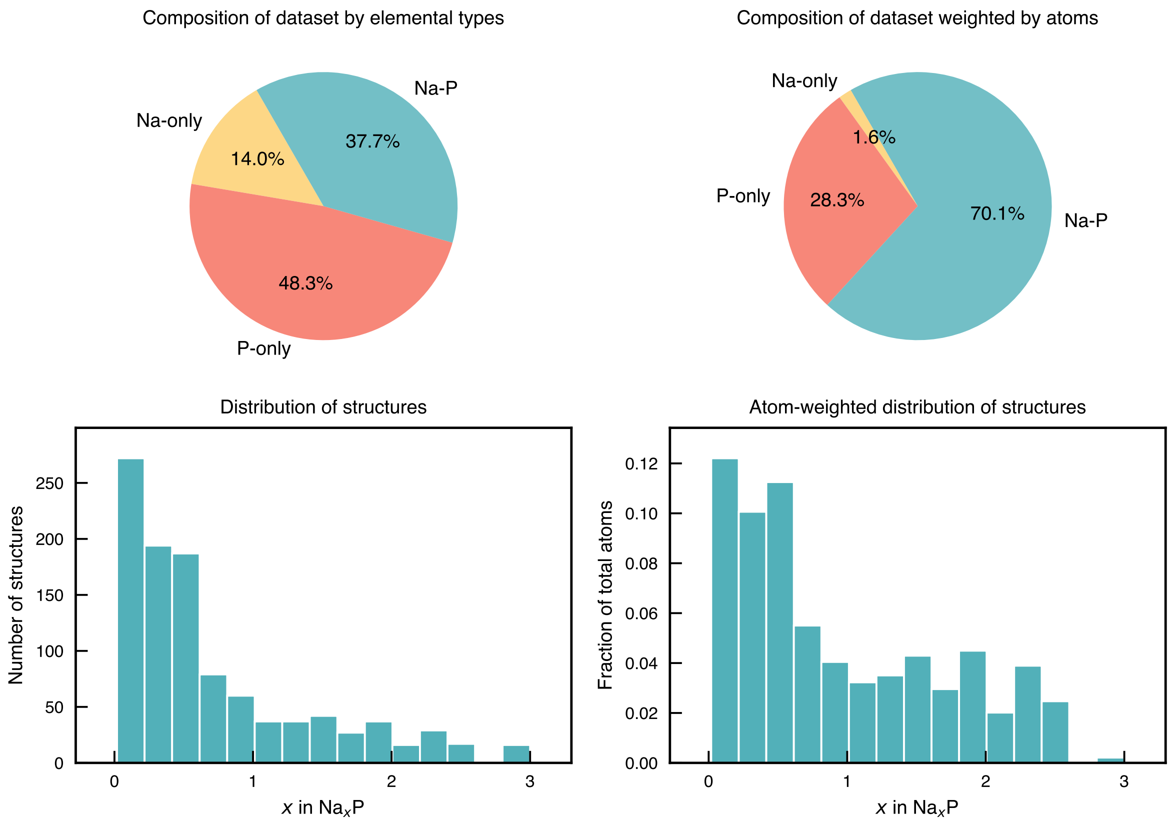

Figure S1: Structures in the Iter-2.2 training set. *Top:* Proportion of Na-only, P-only, and Na–P binary structures, shown by structure count (left) and atom-weighted distribution (right). *Bottom:* Distribution of Na_xP structures (left) and atom-weighted fraction of structures (right) at different Na content.

The composition of the training dataset, categorized by elemental types (e.g., P-only, Na-only, and Na–P binary systems), is illustrated in the upper panel of Figure S1. Since the a-Na_xP structures typically contain more atoms, the atom-weighted distribution is also included on the right. The lower panel of Figure S1 shows the distribution of Na_xP structures based on their Na:P ratios. The underrepresentation of configurations with Na:P ratios above 2.6 can be attributed to three main reasons: (i) no known crystalline Na–P phases exist between NaP and Na_3P; (ii) initial CASTEP geometry optimizations were only performed for a-Na_xP structures with $x < 1.0$; (iii) generating a-Na_xP structures with $x > 2.6$ through iterative MD simulations would require single-point computations of structures with over 390 atoms, which is computationally expensive at the applied level of theory. Nonetheless, subsequent studies confirmed that the MACE model remains sufficiently transferable across the compositional range.

S1.2 MACE training

MACE models were trained using a single RTX 6000 Ada Generation GPU. The dataset was split by assigning 10% of each configuration type to the testing set, and randomly selecting 5% of the remainder training set for validation during training. A vanilla MACE model with a model size of 128 equivariant messages and two message-passing layers was used. Each layer had a correlation order of three, an angular resolution of $l_{\max} = 3$, and a cutoff radius of $r_{\max} = 6.0$ Å. Training was conducted using a standard weighted energy–forces loss function, with loss weights set to $\lambda_E = 1$, $\lambda_F = 100$ for the first 400 epochs, and adjusted to $\lambda_E = 1000$, $\lambda_F = 100$ for the final 350 epochs. The final MACE model achieved root-mean-square errors (RMSE) of 7.7 meV atom^{-1} (0.74 kJ mol^{-1}) for energies and 129.5 meV Å^{-1} for forces on the testing set (Figure S2).

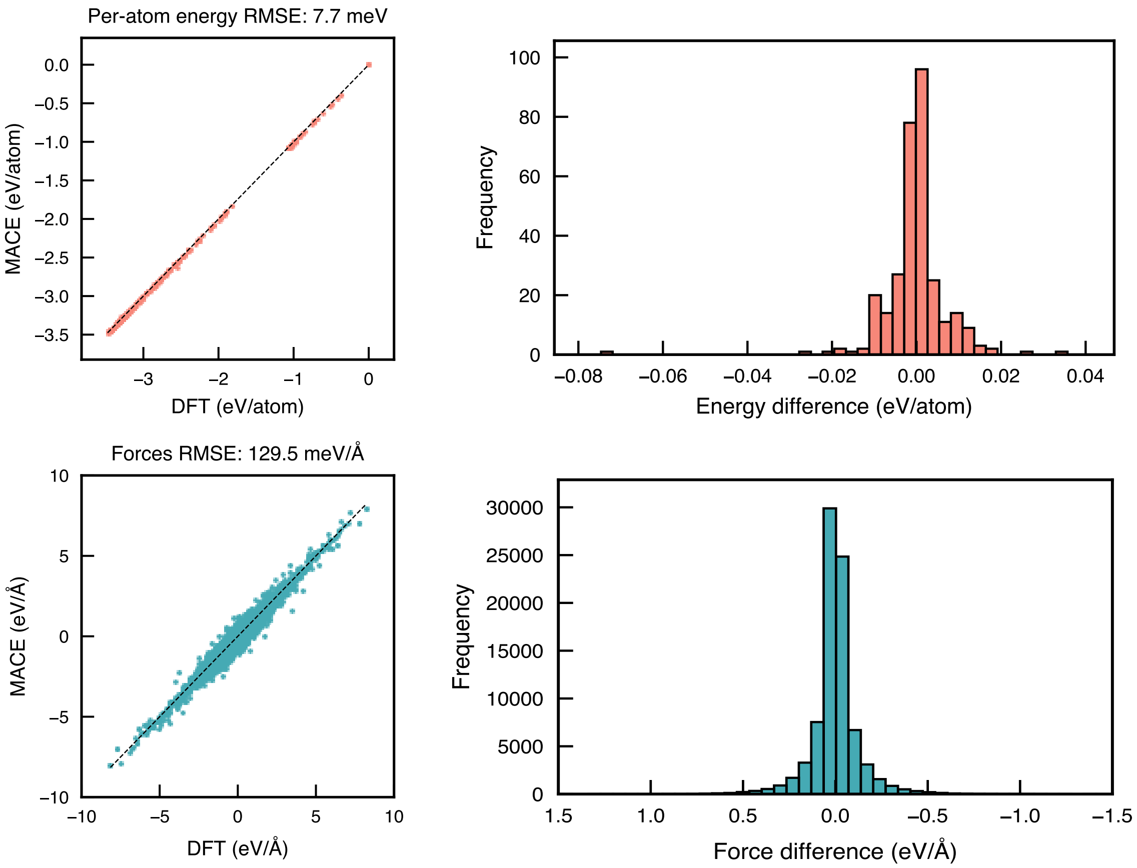

Figure S2: Numerical performance of the Iter-2.2 MACE model. *Left:* Parity plots comparing MACE and DFT energies (top) and forces (bottom) on the testing set. *Right:* Per-structure energy (top) and per-atom force (bottom) differences on the testing set.

S1.3 MD simulations

S1.3.1 Generation of low-density a-P structures

Nine 108-atom a-P cells, along with four low-density variants of each were generated via MD simulations using LAMMPS, as detailed in Section S1.1.1.

S1.3.2 MD for iterative MACE fitting

The iterative generation of a-Na_xP structures was performed using the `MACECalculator` functionality implemented in the ASE package,[S7] as detailed in Section S1.1.2.

S1.3.3 Production MD

Melt–quench. Thirteen a-Na_xP configurations were prepared for structural and energetic analysis. A 248-atom a-P cell was isotropically expanded to densities of 1.99, 1.83, 1.47, 1.27, 1.11, 0.97, 0.87, 0.79, 0.71, 0.65, 0.60, 0.55, and 0.50 g cm^{-3} to accommodate the insertion of 31, 62, 124, 186, 248, 310, 372, 434, 496, 558, 620, 682, and 744 Na atoms, respectively. Each structure was melted at 1,200 K for 10 ps and subsequently quenched to 800 K in the NVT ensemble, using the Nosé–Hoover thermostat[S8,S9] as implemented in ASE. A further quench to 5 K at 1 bar was performed in the NPT ensemble using the Berendsen scheme.[S22] Two quench rates, 10^{13} K s^{-1} and 10^{12} K s^{-1}, were tested, with their effects discussed in Section S2.1. The resulting structures were optimized using the LBFGS algorithm with a force convergence criterion of $f_{max} = 0.01$ eV Å^{-1}.

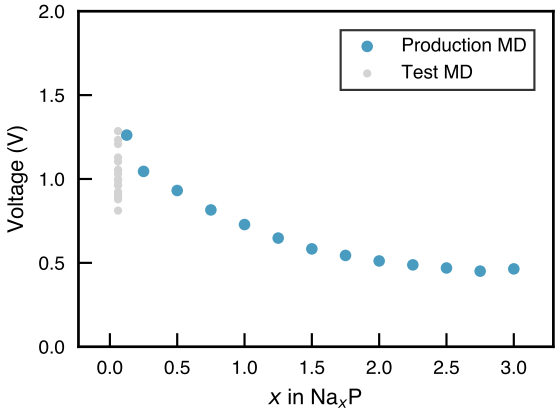

Figure S3: Effect of the MD scheme on structural energies. Grey points at $x = 0.06$ correspond to voltages calculated during initial MD tests of different melt–quench schemes and initial densities, plotted against voltages derived from the final MD scheme.

To determine the optimal MD protocol, we conducted systematic tests on a $Na_{15}P_{248}$ structure. The initial a-P density prior to Na insertion was varied between 1.8, 1.9, 2.0, and 2.1 g cm^{-3}, while the switching temperature for the Berendsen NPT ensemble was tested at 500, 600, 700, and 800 K. Lower initial densities and switching temperatures increased the likelihood of P_4 molecule formation. The grey points in Figure S3 show the corresponding voltage from the test runs, highlighting the sensitivity of structural energy to simulation conditions at low Na content, where numerous insertion sites are available. However, this effect gradually diminished as Na content increased. The final MD scheme described above was selected to maximize voltage while minimizing P_4 formation.

Another set of twelve 108-P a-Na_xP structures was generated following the same protocol, applying only the 10^{12} K s^{-1} quench rate, for LOBSTER analysis (Section S1.5). Figures 1b–d, 2a–b, and 3a present analyses based on the larger structure set, while Figures 1a and 3b–c correspond to the smaller structure set.

High-temperature sodiation and de-sodiation. A 256-atom black P cell was expanded by 124% along the x-axis to create empty space, of which was filled with 256 Na atoms placed randomly using the same hard-sphere cutoff as described in Section S1.1.1. This cell was equilibrated at 600 K and 1 bar for 100 ps in the NPT ensemble using the Berendsen scheme, followed by a linear quench to 5 K over 60 ps. The final configuration was relaxed using the LBFGS algorithm with a force convergence criterion of $f_{\text{max}} = 0.001$ eV Å^{-1}.

De-sodiation was simulated in four sequential steps, each involving the removal of 25% of the total Na content from the relaxed sodiated structure. Atoms were selectively removed starting from those farthest from the originally P-rich region along the x-axis. After each removal, the structure underwent the same annealing, quenching, and optimization procedure to allow for structural reorganization before proceeding to the next extraction step.

A parallel simulation conducted at 500 K exhibited qualitatively similar structural evolution and cluster fragment statistics throughout the de-sodiation process.

S1.4 DFT computations

S1.4.1 CASTEP geometry optimization

Geometry optimizations of the initial Na-inserted a-P and black P (Section S1.1.1) were carried out using DFT implemented in CASTEP 23.1,[S3] with the Perdew–Burke–Ernzerhof (PBE) functional[S23] and TS pairwise dispersion correction.[S10] All computations were performed at the Γ point with a plane-wave energy cutoff of 500 eV, and a SCF convergence threshold of 2×10^{-6} eV. The optimization process used a displacement tolerance of 0.002 Å, an energy tolerance of 2×10^{-5} eV, a force tolerance of 0.05 eV Å^{-1}, and a stress tolerance of 0.1 GPa.

S1.4.2 CASTEP single-point computations

Single-point computations for generating training and testing data labels were computed using the PBE functional. The plane-wave cutoff energy was set to 700 eV, with an SCF convergence threshold of 10^{-8} eV. The Brillouin zone was sampled using a Monkhorst–Pack grid[S24] with a k-point spacing of $2\pi \times 0.04$ Å^{-1}. Core electrons were described using CASTEP on-the-fly pseudopotentials. TS pairwise dispersion correction were used for Iter-1 models but subsequently removed due to reasons described in Section S1.1.3. The energies of all reference crystalline phases considered in the analysis were computed at a consistent level of theory.

S1.4.3 VASP single-point computations

GAP-RSS labelling. Single-point computations during the GAP-driven RSS process (Section S1.1.3) were performed using the projector augmented-wave (PAW) method[S25] in VASP[S20] with the PBE exchange–correlation functional.[S23] A plane-wave energy cutoff of 700 eV and an electronic convergence criterion of 10^{-7} eV were used. The Brillouin zone was sampled using an automatically generated k-point mesh with a spacing of 0.2 Å^{-1}.

Electronic-structure computations for LOBSTER. The PBE functional was used with a plane-wave energy cutoff of 520 eV and an SCF energy convergence threshold of 10^{-5} eV. Convergence tests at tighter criteria of 10^{-6} and 10^{-7} eV showed no improvement in the calculated charges within the numerical precision of the LOBSTER output. Gaussian smearing with a width of 0.05 eV was applied. An explicit k-point mesh with 8 points was used to sample the Brillouin zone in all cells.

S1.5 Charge and DOS computations

Atomic charges were evaluated using Löwdin population analysis, and electronic densities of states (DOS) were derived from the self-consistent wavefunctions by analytical projection onto a local auxiliary basis of valence *s* and *p* orbitals, as implemented in the Local-Orbital Basis Suite Towards Electronic-Structure Reconstruction (LOBSTER).[S26–S29] A default minimal basis set, including Na 3s and P 3s, 3p orbitals, was employed. The analysis was performed on single-point wavefunctions obtained from VASP calculations (Section S1.4.3).

Compared with other charge-partitioning schemes, such as the density-based Bader method, the orbital-based Löwdin approach was adopted because it is computationally efficient and avoids unphysical charge assignments that were reported for Na and Li in nanoporous carbon systems.[S30,S31]

S1.6 Computational costs

The final MACE model was trained on 2,783 DFT-labelled structures, representing systems ranging in size from fewer than ten to over four hundred atoms. Thus, the computational time for the DFT labelling varied from a few minutes on a 128-core CPU node to over 20 hours using up to four 128-core nodes on the UK National Supercomputing Service (ARCHER2). Although the total wall time is difficult to quantify precisely, the DFT labelling stage represented by far the most resource-intensive component of this study.

Subsequent model training and MD simulations were performed on a single NVIDIA A100 GPU. During the iterative training process, the time per epoch increased as the training set expanded. For the final model, using a batch size of 10, each epoch required approximately 9.4 minutes. The optimal model checkpoint was saved after 84 epochs, with additional epochs carried out to ensure full convergence. Once trained, a 100 ps (100,000-timestep) simulation of a 400-atom amorphous Na–P model required approximately 1.5 GPU hours using the `MACECalculator` implemented in ASE.

S2 Supplementary results and discussions

S2.1 Effects of quench rates

As mentioned in Section S1.3.3, two quench rates — 10^{13} and 10^{12} K s^{-1} — were tested during the melt–quench generation of a-Na_xP structures. As shown in Figure S4, structures generated with the faster quench exhibit slightly higher normalized formation energies compared to their slow-quenched counterparts. The energy difference generally diminishes with increasing Na content, except in the case of a-Na_3P where the slow-quenched structure demonstrates markedly enhanced stabilization.

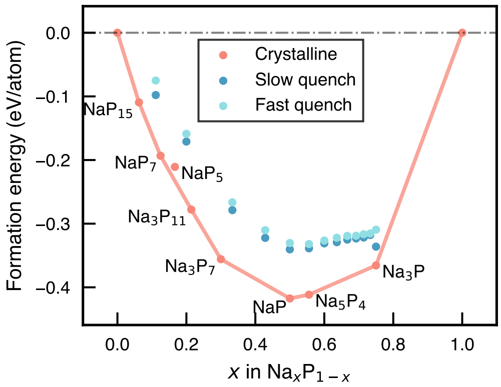

Figure S4: Convex hull including the formation energies of fast-quenched a-Na_xP (light blue).

Despite the reduced thermodynamic stability, our fast-quenched samples also conform well with the Zintl–Klemm formalism. As illustrated in Figure S5, the P–P coordination distributions closely resemble those shown in Figure 2a and b. However, the fast-quenched structures

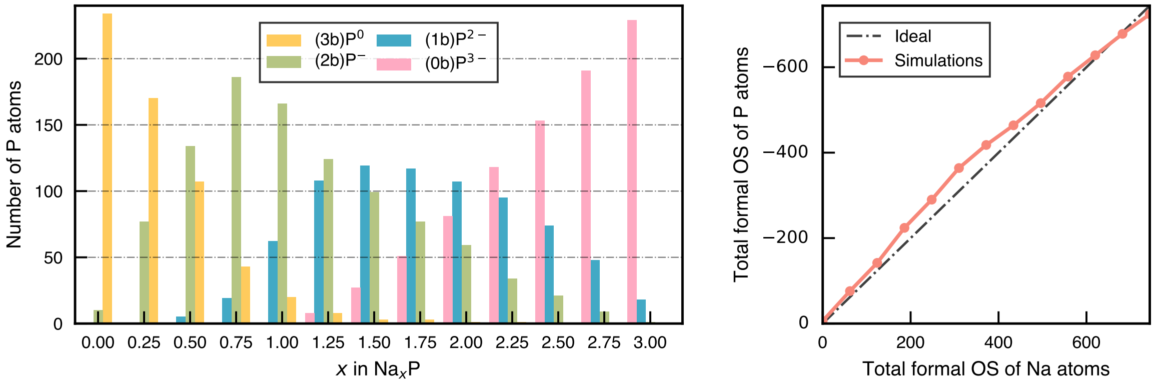

Figure S5: Coordination analysis of the fast-quenched samples. *Left:* Distribution of P atoms with different homoatomic coordination numbers. *Right:* Total formal oxidation state (OS).

exhibit a modest increase in the number of (1b)P^{2-} species, accompanied by a slight decrease in P atoms of other coordination types, consistent with the observation that (1b)P^{2-} units are thermodynamically disfavoured.

S2.2 P–P bond lengths

Nearest-neighbour P–P distance distributions were calculated for P with different homonuclear connectivity, and are shown in the left panel of Figure S6. As expected from steric considerations, (3b)P^0 atoms exhibit, on average, longer P–P bonds than (2b)P^- ones. However, the (1b)P^{2-} ions deviate from the steric expectation, exhibiting both a larger average than (2b)P^-, and a larger spread in bond lengths, as indicated by their higher standard deviation.

To better understand this anomaly, we analyzed the influence of neighbouring Na atoms (within a 3.4 Å cutoff) on the P–P bond lengths of the (1b)P^{2-} species. The violin plot on the bottom right of Figure S6 shows that, for the (1b)P^{2-} species, an increasing number of nearby Na atoms correlates with larger average and maximum nearest-neighbour P–P distances. This bond lengthening effect may arise from electron transfer from adjacent Na atoms, which could weaken the P–P interactions through increased electron density localization on the P sites.

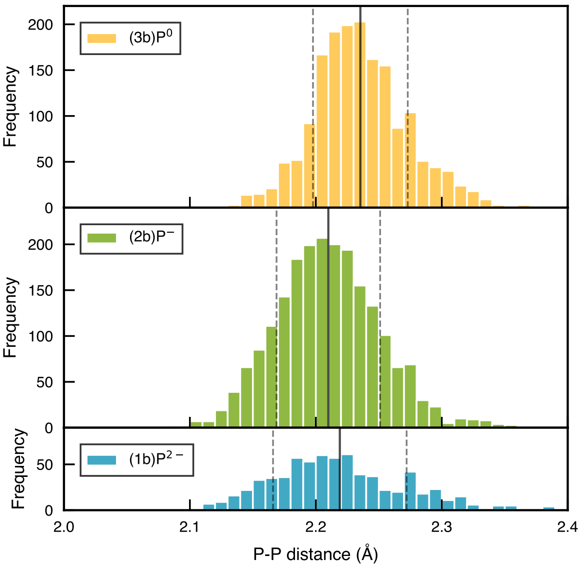

Coordination	Mean (Å)
(3b)P^0	2.24 ± 0.038
(2b)P^-	2.21 ± 0.041
(1b)P^{2-}	2.22 ± 0.053

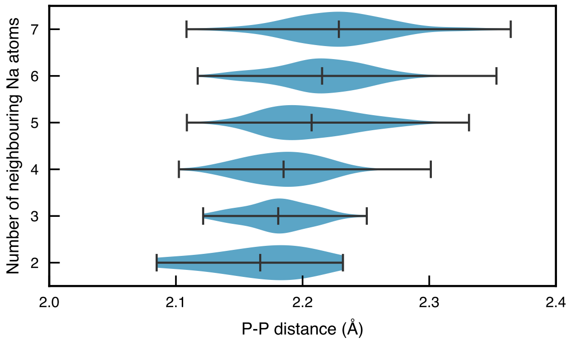

Figure S6: P–P bond length analysis. *Left:* P–P bond length (cutoff = 2.4 Å) distributions, resolved by P connectivity. Solid and dotted lines indicate mean and standard deviation. Summary statistics are shown in the table at the top right. *Bottom right:* Correlation between the number of Na atoms within a 3.4 Å cutoff and the P–P bond lengths of the (1b)P^{2-} species.

S2.3 Atomic and environmental energies of Na atoms

The MACE-predicted atomic energies of Na atoms in a-Na_xP, categorized by the number of neighbouring P atoms within a 3.4 Å cutoff, are shown in the left panel of Figure S7. A clear stabilization trend is observed as the number of neighbouring P atoms increases, possibly implying stronger local ionic interactions in more P-rich environments.

The right panel of Figure S7 shows the corresponding local environmental energies, defined as the sum of the central Na atom energy and energies of neighbouring P atoms within the same cutoff distance. Interestingly, the average environmental energy across different Na coordination environments remains close to 0 eV, suggesting that the local environments around Na atoms are energetically compensated for by nearby P atoms. The interpretation of this energetic balance warrants further investigation.

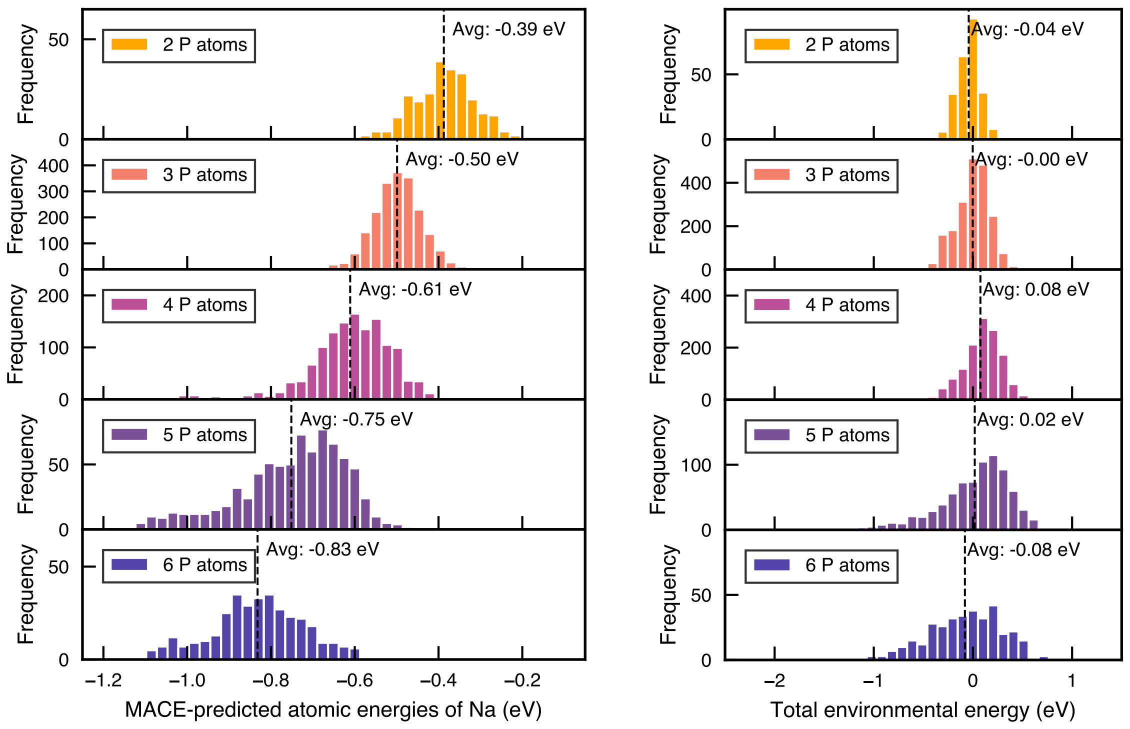

Figure S7: Local energy analysis of Na in a-Na_xP. *Left:* MACE atomic energies of Na atoms, grouped by the number of neighbouring P atoms within a 3.4 Å cutoff. Energies are referenced to bulk body-centered cubic Na. *Right:* Local environmental energies of Na atoms, calculated as the sum of the Na atom energy and energies of neighbouring P atoms within the same cutoff.

S2.4 Atomic energies versus charges

To better visualize the correlation between atomic energy and charge, the MACE energies of individual atoms are plotted against their respective charges and shown in Figure S8. The distribution of P atom energies mirrors the trends observed in Figure 3, with (1b)P^{2-} species exhibiting the highest average energy among all P coordination classes. Distinct charge separation is evident across different P species, where decreasing homoatomic connectivity correlates with increasingly negative Löwdin charges.

In contrast, Na atoms have a more continuous relationship between energy and charge, where Na atoms with a greater number of neighbouring P atoms (purple) tend to exhibit more positive Löwdin charges and lower MACE atomic energies. This trend highlights the role of local Na–P coordination in stabilizing Na atoms: the more an Na atom is embedded within the P network, the greater the charge transfer. The resulting stronger ionic interactions are then reflected in the lower atomic energy.

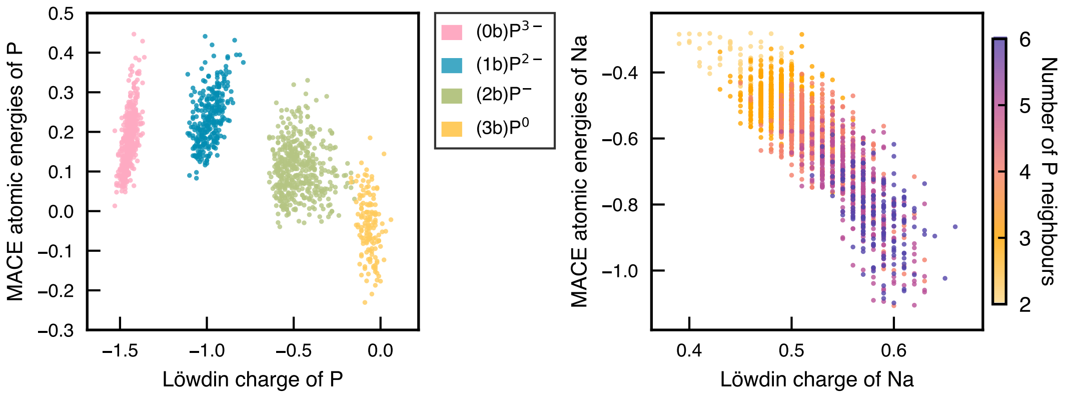

Figure S8: MACE atomic energies against Löwdin charges for individual P (left) and Na (right) atoms in a-Na_xP structures. P atoms are colour-coded based on local homonuclear connectivity; Na atoms are colour-coded according to the number of neighbouring P atoms within a 3.4 Å cutoff.

S2.5 Electronic structure evolution with Na filling

The electronic partial density of states (PDOS) of Na and P at different stages of Na filling were extracted from the same set of LOBSTER calculations used for the Löwdin charge analysis and are shown in Figure S9. Initially, Na atoms transfer their valence electrons almost completely to the P framework, as the Na 3s orbital remains unoccupied above the Fermi level, E_F. With increasing Na content, occupied states emerge with a finite PDOS at E_F. A similar ionic-to-metallic crossover was reported previously for porous-carbon-confined Na atoms.[S30]

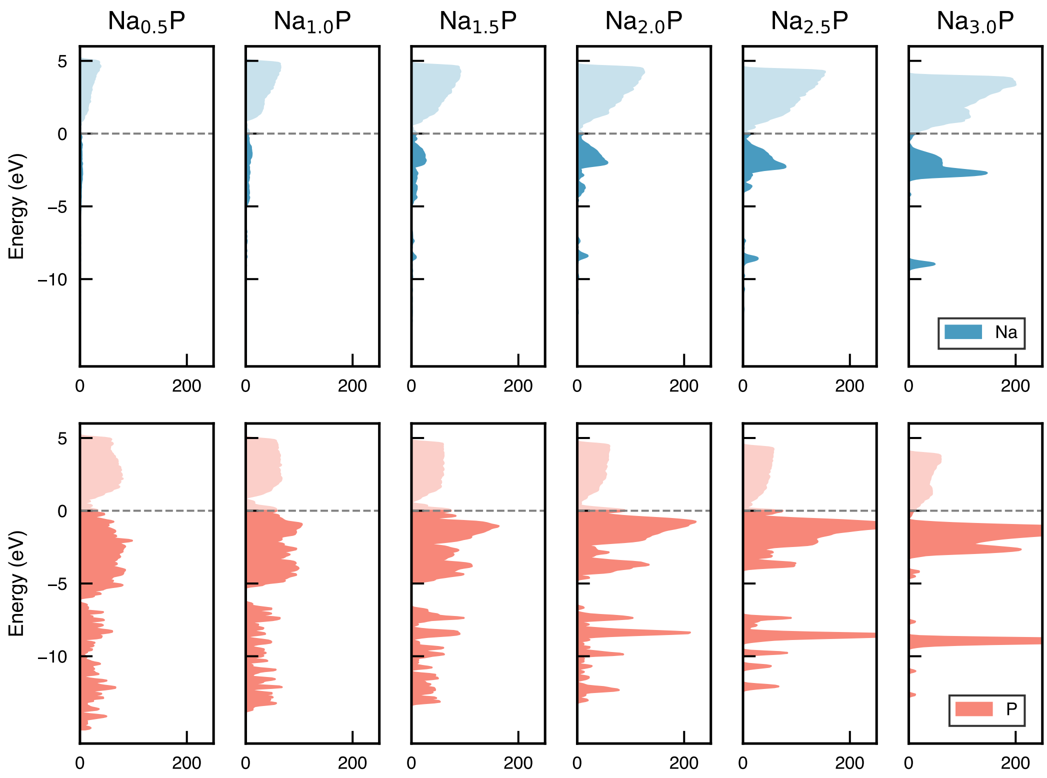

Figure S9: Evolution of the PDOS for a-Na_xP structures with increasing Na content, showing Na contributions projected onto 3s orbitals (top) and P contributions projected onto 3s and 3p orbitals (bottom).

P initially shows a finite PDOS at E_F, likely arising from localized states associated with structural disorder and defects. The broad 3s and 3p bonding bands at this stage indicate substantial orbital overlap within the covalent P network. As Na content increases and the P atoms become progressively more isolated, these bands narrow significantly due to reduced orbital interactions. The concurrent decrease in valence-state PDOS suggests that electrons transferred from Na occupy previously empty P 3p orbitals, stabilizing the overall electronic structure.

S2.6 Ring and cluster fragment counts during de-sodiation

Ring and cluster analysis during de-sodiation simulations was performed using a custom Python script to track local structural evolution. As shown in Figure S10, progressive Na extraction led to a steady increase in the number of primitive five-membered rings and local cluster fragments, specifically P3]P2[P3 and P2[P3]P2 (illustrated on the right). In contrast, the number of six-membered rings, characteristic of crystalline black P, increased only marginally. This asymmetry in the recovery of structural motifs suggests that the structural disorder introduced during sodiation is largely irreversible, with the de-sodiated structure tending toward locally

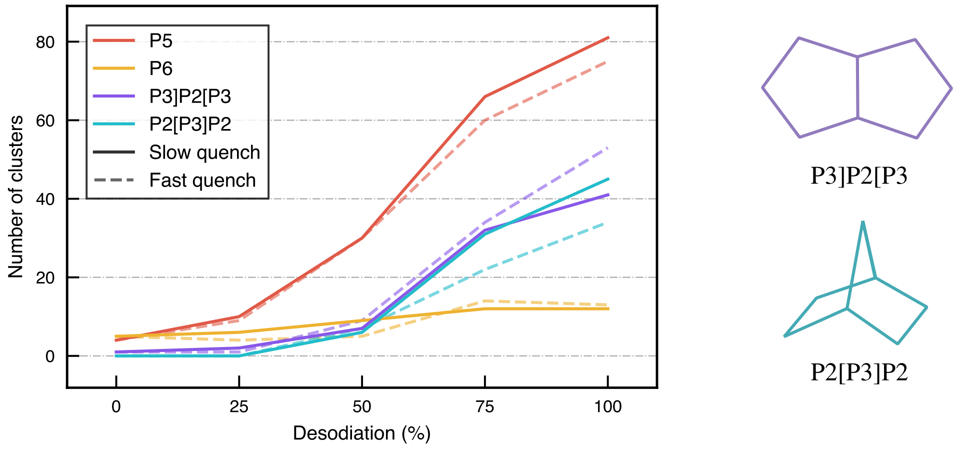

Figure S10: Number of rings and local cluster fragments after 25%, 50%, 75%, and 100% de-sodiation at different quench rates. Illustrations of P3]P2[P3 and P2[P3]P2 cluster fragments (cf. Ref. S32) are shown on the right.

disordered amorphous motifs.

To examine the effect of quench rates on the evolution of the disordered network topology, the de-sodiated structures were equilibrated at quench rates of 10^{12} K s^{-1} and 10^{13} K s^{-1}. As shown by the comparison between the solid and dotted lines in Figure S10, both cases show a consistent preference for P5 over P6 rings, indicating no tendency toward crystalline recovery at a quench rate an order of magnitude slower. A slight increase in the relative abundance of $P_2[P_3]P_2$ compared to $P_3]P_2[P_3$ is observed at the slower quench rate, although this variation is small and likely stochastic in nature.

S2.7 Effects of many-body dispersion corrections

As discussed in Section S1.1.3, dispersion corrections were excluded from Iter-2.0 onward, as their influence became increasingly unclear in a dataset dominated by ionic structures. Nevertheless, previous studies have emphasized the importance of dispersion corrections in accurately describing elemental P systems.[S1,S11,S12] Since the fully de-sodiated structure is an elemental P system, we evaluated the potential impact of dispersion corrections on those structures by repeating the same simulation protocol using the P-GAP-20 model. This model had been trained at the PBE+MBD level of theory and incorporates a $1/r^6$-dependent baseline to account for long-range interactions.[S1]

After holding at 600 K and 1 bar for 100 ps, and quenching at 10^{13} K s^{-1} (Section S1.3.3), the final structure obtained with the P-GAP-20 model was noticeably more contracted than

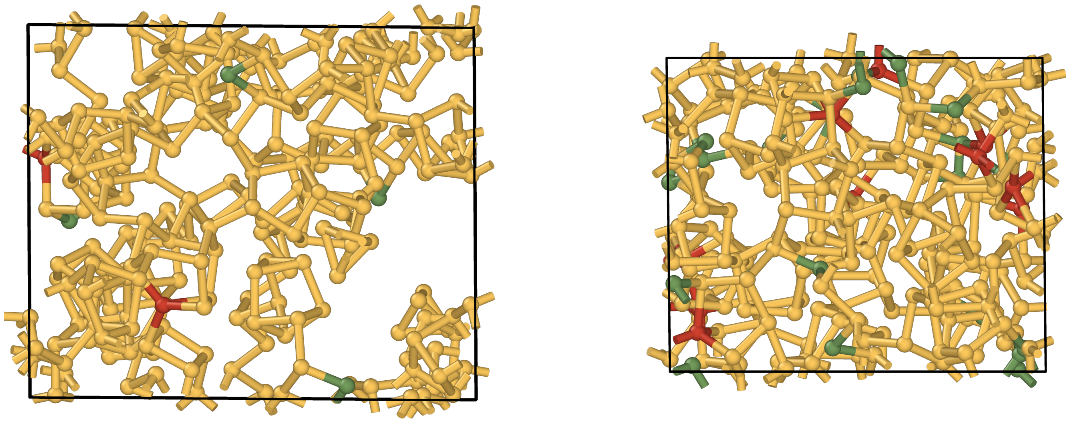

Figure S11: Final structures of the fully de-sodiated cell after relaxation using the MACE model (left) and the P-GAP-20 model from Ref. S1 (right). Cell sizes are shown to scale.

that obtained with the Na–P MACE model of the present work, consistent with the absence of long-range dispersion corrections in the training labels of the latter.

Despite the large difference in final density — 1.47 versus 2.46 g cm^{-3} — both models produced similar local structural features, as reflected in the comparable fragment counts in Table S3. This suggests that the MACE model remains robust in reproducing local bonding environments even at expanded volumes. We therefore attribute the expansion observed in the MACE-relaxed structure to limitations in the training labels rather than to a physically meaningful effect.

Table S3: Comparison of density and fragment counts of fully de-sodiated structures obtained from the Na–P MACE model of the present work and the elemental P-GAP-20 model.[S1]

ML models	Density (g cm^{-3})	Fragment counts			
		**P5**	**P6**	**P3]P2[P3**	**P2[P3]P2**
Na–P MACE	1.47	75	13	53	34
P-GAP-20	2.46	75	20	52	39

S2.8 Structural fragments in related crystalline phases

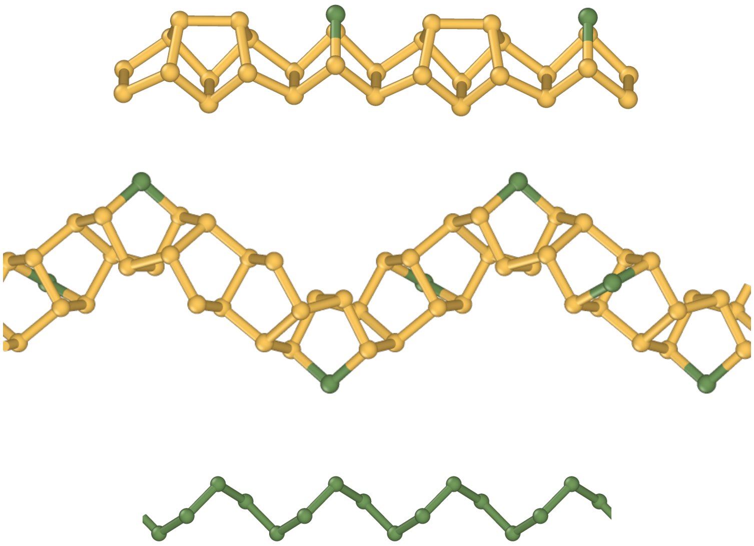

Figure S12: P structural framework in crystalline NaP_{15} (top), NaP_7 (middle) and NaP (bottom), shown for comparison with Figure 2c. In NaP_{15}, ${}^1_\infty(P_{15}^-)$ tubes form through the polymerization of alternating P_8 (similar to the As_4S_4 molecular structure) and P_7^- (a norbornane derivative) units. In NaP_7, ${}^1_\infty(P_7^-)$ helical tubes form by connecting norbornane-like P_7^- clusters via some of the base and the bridging P atoms. The anionic partial framework in NaP consists of ${}^1_\infty(P^-)$ helical chains.

S3 Supplementary references

[S1] V. L. Deringer, M. A. Caro, G. Csányi, *Nat. Commun.* **2020**, *11*, 5461.

[S2] A. P. Thompson, H. M. Aktulga, R. Berger, D. S. Bolintineanu, W. M. Brown, P. S. Crozier, P. J. in 't Veld, A. Kohlmeyer, S. G. Moore, T. D. Nguyen, R. Shan, M. J. Stevens, J. Tranchida, C. Trott, S. J. Plimpton, *Comput. Phys. Comm.* **2022**, *271*, 108171.

[S3] S. J. Clark, M. D. Segall, C. J. Pickard, P. J. Hasnip, M. I. J. Probert, K. Refson, M. C. Payne, *Z. Kristallogr.* **2005**, *220*, 567.

[S4] A. Jain, S. P. Ong, G. Hautier, W. Chen, W. D. Richards, S. Dacek, S. Cholia, D. Gunter, D. Skinner, G. Ceder, K. A. Persson, *APL Mater.* **2013**, *1*, 011002.

[S5] C. J. Pickard, R. J. Needs, *Phys. Rev. Lett.* **2006**, *97*, 045504.

[S6] C. J. Pickard, R. J. Needs, *J. Phys.: Condens. Matter* **2011**, *23*, 053201.

[S7] A. H. Larsen, J. J. Mortensen, J. Blomqvist, I. E. Castelli, R. Christensen, M. Dułak, J. Friis, M. N. Groves, B. Hammer, C. Hargus, E. D. Hermes, P. C. Jennings, P. B. Jensen, J. Kermode, J. R. Kitchin, E. L. Kolsbjerg, J. Kubal, K. Kaasbjerg, S. Lysgaard, J. B. Maronsson, T. Maxson, T. Olsen, L. Pastewka, A. Peterson, C. Rostgaard, J. Schiøtz, O. Schütt, M. Strange, K. S. Thygesen, T. Vegge, L. Vilhelmsen, M. Walter, Z. Zeng, K. W. Jacobsen, *J. Phys.: Condens. Matter* **2017**, *29*, 273002.

[S8] S. Nosé, *Mol. Phys.* **1984**, *52*, 255.

[S9] W. G. Hoover, *Phys. Rev. A* **1985**, *31*, 1695.

[S10] J. P. Perdew, K. Burke, M. Ernzerhof, *Phys. Rev. Lett.* **1996**, *77*, 3865.

[S11] F. Bachhuber, J. von Appen, R. Dronskowski, P. Schmidt, T. Nilges, A. Pfitzner, R. Weihrich, *Angew. Chem. Int. Ed. Engl.* **2014**, *53*, 11629.

[S12] G. Sansone, A. J. Karttunen, D. Usvyat, M. Schütz, J. G. Brandenburg, L. Maschio, *Chem. Commun.* **2018**, *54*, 9793.

[S13] T. Bučko, S. Lebègue, J. Hafner, J. G. Ángyán, *Phys. Rev. B* **2013**, *87*, 064110.

[S14] M. Mayo, K. J. Griffith, C. J. Pickard, A. J. Morris, *Chem. Mater.* **2016**, *28*, 2011.

[S15] V. L. Deringer, C. J. Pickard, G. Csányi, *Phys. Rev. Lett.* **2018**, *120*, 156001.

[S16] N. Bernstein, G. Csányi, V. L. Deringer, *npj Comput. Mater.* **2019**, *5*, 1.

[S17] Y. Liu, J. D. Morrow, C. Ertural, N. L. Fragapane, J. L. A. Gardner, A. A. Naik, Y. Zhou, J. George, V. L. Deringer, An automated framework for exploring and learning potential-energy surfaces **2024**, arXiv:2412.16736 [physics.comp-ph].

[S18] A. P. Bartók, R. Kondor, G. Csányi, *Phys. Rev. B* **2013**, *87*, 184115.

[S19] M. W. Mahoney, P. Drineas, *Proc. Natl. Acad. Sci. U. S. A.* **2009**, *106*, 697.

[S20] G. Kresse, J. Furthmüller, *Phys. Rev. B* **1996**, *54*, 11169.

[S21] A. P. Bartók, M. C. Payne, R. Kondor, G. Csányi, *Phys. Rev. Lett.* **2010**, *104*, 136403.

[S22] H. J. C. Berendsen, J. P. M. Postma, W. F. van Gunsteren, A. DiNola, J. R. Haak, *J. Chem. Phys.* **1984**, *81*, 3684.

[S23] J. P. Perdew, K. Burke, M. Ernzerhof, *Phys. Rev. Lett.* **1996**, *77*, 3865.

[S24] H. J. Monkhorst, J. D. Pack, *Phys. Rev. B* **1976**, *13*, 5188.

[S25] G. Kresse, D. Joubert, *Phys. Rev. B* **1999**, *59*, 1758.

[S26] S. Maintz, V. L. Deringer, A. L. Tchougréeff, R. Dronskowski, *J. Comput. Chem.* **2016**, *37*, 1030.

[S27] C. Ertural, S. Steinberg, R. Dronskowski, *RSC Adv.* **2019**, *9*, 29821.

[S28] R. Nelson, C. Ertural, J. George, V. L. Deringer, G. Hautier, R. Dronskowski, *J. Comput. Chem.* **2020**, *41*, 1931.

[S29] S. Maintz, V. L. Deringer, A. L. Tchougréeff, R. Dronskowski, *Journal of Computational Chemistry* **2013**, *34*, 2557.

[S30] V. L. Deringer, C. Merlet, Y. Hu, T. H. Lee, J. A. Kattirtzi, O. Pecher, G. Csányi, S. R. Elliott, C. P. Grey, *Chemical Communications* **2018**, *54*, 5988.

[S31] C. Ertural, R. P. Stoffel, P. C. Müller, C. A. Vogt, R. Dronskowski, *Chemistry of Materials* **2022**, *34*, 652.

[S32] S. Böcker, M. Häser, *Z. Anorg. Allg. Chem.* **1995**, *621*, 258.
